# Running Worms: *C. elegans* Self-Sorting by Electrotaxis

**DOI:** 10.1371/journal.pone.0016637

**Published:** 2011-02-04

**Authors:** Xavier Manière, Félix Lebois, Ivan Matic, Benoit Ladoux, Jean-Marc Di Meglio, Pascal Hersen

**Affiliations:** 1 Laboratoire TaMaRa, U1001 Université Paris Descartes and INSERM, Paris, France; 2 Laboratoire Matière et Systèmes Complexes, UMR7057 Université Paris Diderot and CNRS, Bâtiment Condorcet, Paris, France; Buck Institute for Age Research, United States of America

## Abstract

The nematode *C. elegans* displays complex dynamical behaviors that are commonly used to identify relevant phenotypes. Although its maintenance is straightforward, sorting large populations of worms when looking for a behavioral phenotype is difficult, time consuming and hardly quantitative when done manually. Interestingly, when submitted to a moderate electric field, worms move steadily along straight trajectories. Here, we report an inexpensive method to measure worms crawling velocities and sort them within a few minutes by taking advantage of their electrotactic skills. This method allows to quantitatively measure the effect of mutations and aging on worm's crawling velocity. We also show that worms with different locomotory phenotypes can be spatially sorted, fast worms traveling away from slow ones. Group of nematodes with comparable locomotory fitness could then be isolated for further analysis. *C. elegans* is a growing model for neurodegenerative diseases and using electrotaxis for self-sorting can improve the high-throughput search of therapeutic bio-molecules.

## Introduction

The nematode *Caenorhabditis elegans* [1] is routinely used as a model organism to investigate key biological processes including aging [2]–[4], functioning of the neural system [5], and muscle degeneration [6] to cite but a few. Its genetic and phenotypic traits are extremely well documented [1]. Moreover, a comprehensive library of mutants is available [7] and powerful tools, such as RNAi, allow manipulation of gene expression. The locomotion abilities and the dynamical behaviors of worms provide important displays of their phenotype/genotype and can thus be used as powerful proxies for quantitative analysis. For instance, multiple drugs – *e.g.* those affecting synaptic transporters such as serotonin [8] – and chemicals – *e.g.* those involved in chemotaxis [9] – are known to affect the behavior of worms. Morphological abnormalities – *e.g.* long, dumpy or roller mutants – and neural deficiency – *e.g.* uncoordinated mutants – also correlate with a more or less severely impaired locomotion [1], [5]. In practice, screening for a phenotype of interest, such as abnormal locomotion, is done by visual scoring followed by manual selection. For example, behavioral classes of motility are still the standard way to evaluate the locomotor abilities of *C. elegans*. This is time consuming and hardly quantitative.

Several image-based tracking softwares have been developed to automatically extract locomotion properties of freely crawling worms [10]–[13]. However, freely moving worms have highly unsteady kinematics – worms typically switch between active foraging and resting periods – and their trajectories are complex, rendering a quantitative description difficult. Moreover, the number of analyzed worms cannot be too large to allow for unambiguous worm identification. Other devices, such as worm sorters, are dedicated to high-throughput screening. They are expensive and sort worms only according to a static phenotype (*e.g.* their shape or the expression level of a reporter gene). Recently, an *in vivo* high-throughput microfluidic worm sorter was designed by Rohde *et al.* [14]. Worms were sequentially immobilized one at a time thanks to a pressure controlled valve, analyzed by fluorescence microscopy, released and dispatched to the appropriate exit. Although such a worm sorter is an excellent strategy for high-throughput screening, it requires a high degree of expertise and is, unfortunately, not applicable to analyze locomotion patterns since it deals with mechanically immobilized worms. In this article, we describe an elementary method that combines a direct measurement of the velocity of single worms and the ability to sort multiple worms according to their locomotory skills.

## Results

**Figure 1:**
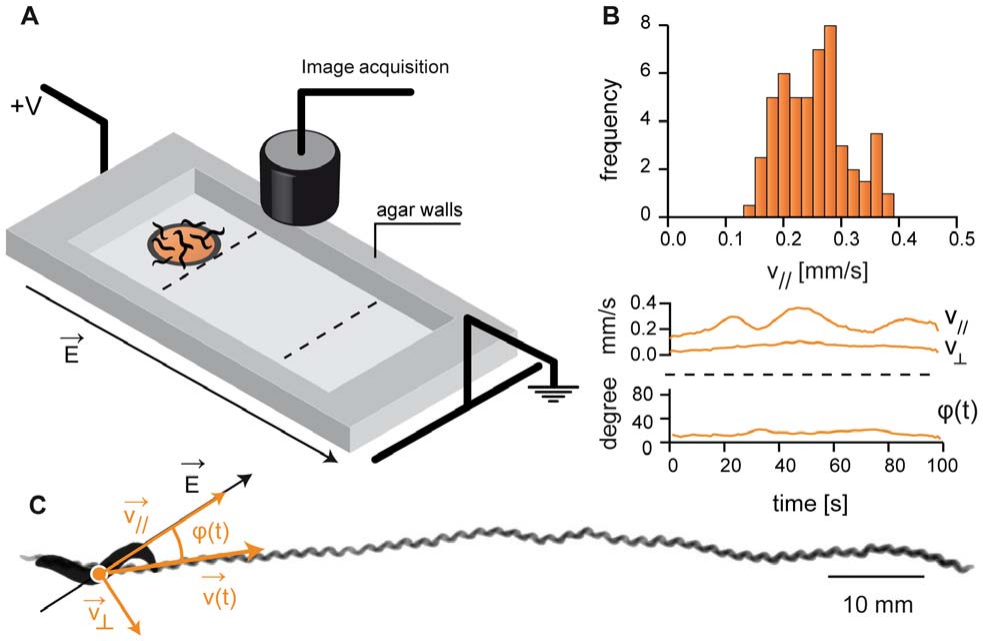
Experimental setup. (A) The setup combines a classic electrophoresis box (∼20 cm long) with a video camera and a LED ring to record images of nematodes moving at the surface of an agar gel. The gel is flat and has walls (in grey) to prevent buffer inflow in the electrotaxis area. (B) Velocity distribution during an electrotactic event and evolution with time of the velocity and the orientation of the trajectory of a single worm performing electrotaxis. This shows that during electrotaxis, a single worm moves steadily in a relatively constant direction. (C) The corresponding trajectory is relatively straight and has an angle θ of 15° with the electric field orientation. The characteristic sinusoidal shape of the nematode crawling gait can be observed, indicating that the worm is moving by generating a rearward flexural wave on its body.

**Figure 2:**
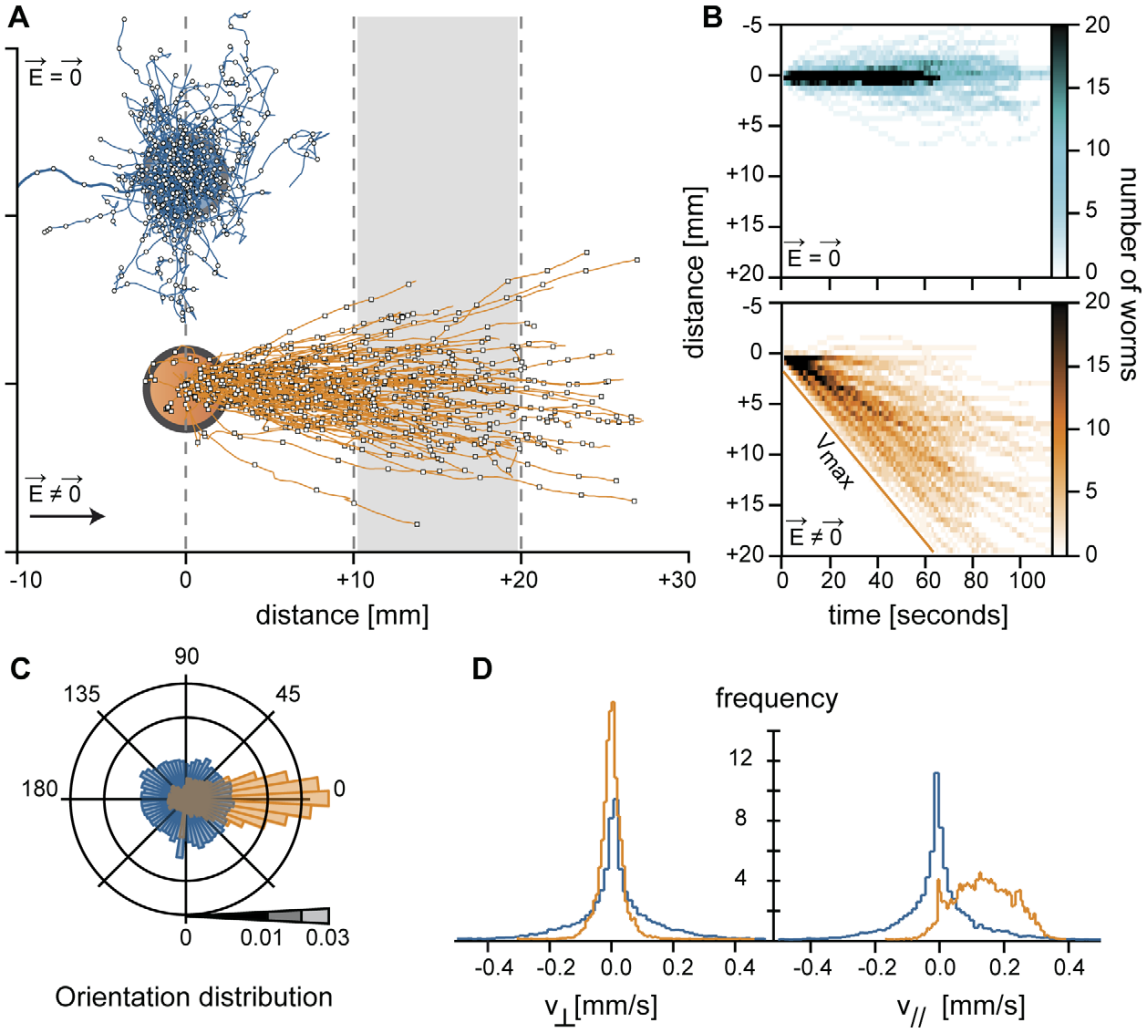
Electrotaxis and directed locomotion. (A) Trajectories obtained from several distinct experiments done with 10–15 worms are displayed on the same graph. Directed locomotion by electrotaxis (orange, N = 130) is observed for a difference of potential of 120 V, while without any electric field, trajectories are randomly oriented (blue, N = 146). (B) From those trajectories, one can extract a spatio-temporal diagram of the density of nematode (graded in orange or blue intensity) at the surface of the gel. Electrotaxis leads to a directed spreading at the surface of the gel. Note that even in a synchronized population of worms there is a large variability in velocity. (C) Orientations of the trajectory are mostly parallel to the electric field (orange), though they vary from one worm to another. It is not known yet how the electrotaxis orientation is set by worms without electrotaxis, trajectories do not exhibit any preferred orientation (blue). (D) The histograms of parallel and perpendicular velocity with (orange, v_//_ = 140±90 µm/s) or without (blue, v_//_ = 1±70 µm/s) an electric field. The measured velocities may depend on environmental conditions such as the presence or absence of nutrients. This is why worms were systematically rinsed in M9 buffer before their transfer. Similarly, the poro-elastic properties and humidity of the agar gel can affect the worms velocity. It is therefore recommended to run a control with wild-type worms to set a reference speed.

Our method is based on the electrotactic ability of *C. elegans* [15], [16]. As first evidenced by Sukul *et al.* [15], *C. elegans* can detect the presence of an electric field. If this field is larger than typically 3 V/cm [16] worms move steadily in the direction of decreasing potentials (Fig. 1 and Fig. 2). Gabel *et al.* evidenced that mutations such as *che-13* and *che-2* and laser ablation that disrupt the functions of amphid sensory neurons also disrupt electrotaxis. Yet, *C. elegans* electro-sensory navigation is still not well understood. Nevertheless, such a robust behavior opens the possibility to sort population of worms. Here, we combined a classic DNA-electrophoresis box (see Fig. 1 and Methods) with a LED ring, for proper illumination, and a video camera to create an inexpensive worm-sorter platform. In a typical experiment, one or several worms are transferred on an agar gel placed in the electrophoresis chamber which is filled with an electrophoresis buffer. The agar pad is typically ten centimeters long, flat and has walls to prevent buffer inflow. As we will discuss next, this elementary setup was sufficient to get reproducible electrotactic runs.

### Quantitative electrotaxis

Figure 2 shows how a group of wild-type worms (N2 strain) spread over the gel surface in function of time with or without an electric stimulation. In absence of applied electric field, worms displayed complex locomotion patterns with reorientations, “omega” bends, reversals, backward motions and pauses. As shown on Figure 2, the resulting trajectories were not oriented (Fig. 2A). Worms only slowly invaded the surface of the agar gel (Fig. 2B), with no preferred movement orientations (Fig. 2C). This can also be seen on the histograms of the components of the velocity perpendicular, v_⊥_, and parallel, v_//_, to the long axis of the elelectrophoresis chamber, which were found to be centred on 0 (Fig. 2D). In contrast, during an electrotactic run, a wild-type worm moved steadily in a well defined direction (Fig. 1B, 1C and Fig. 2; Movie S1). This is the signature of directed locomotion: there were very few events of slow, hesitating forward or backward motion. Repeating this experiment with several worms (>100) showed that all young adult worms were responsive to a difference of potential of 120 V applied to the electrophoresis box. They displayed straight trajectories oriented in average along the electric field direction (Fig. 1, Fig. 2A and 2C). For a given worm, the trajectory orientation remained surprisingly constant on the entire length of the gel (5 cm ∼50 times a young adult worm length) (Fig. 1 and Fig. 2C). Accordingly, the histogram of v_⊥_ was centered on 0, while the histogram of the velocity component parallel to the electric field direction, v_//_, was shifted towards positive velocity, with an average value of 140 µm/s in good agreement with previously reported measurements [10] (Fig. 2D). Only on rare occasions, worms got confused and operated an omega loop before resuming their motion (Movie S2). Increasing the difference of potential from 100 V to 250 V did not affect the worms speed. This means that worms are forced to move by the presence of an electric field but not moved by it, as DNA is by electrophoresis. When suddenly reversing the electric field intensity, worms display a typical omega loop (Movie S3) before they resume their trajectory, evidencing that worms are indeed sensing the existence of an electric field an adjusting its locomotion to it. However, as observed by Gabel *et al.* [16], the trajectories were inclined with respect to the electric field orientation. They reported that the larger the electric field, the larger the angle between the trajectory and the electric field orientation. In our setup, working with 120 V ensured almost parallel trajectories (Fig. 2C). Therefore, electrotaxis appears as an efficient way to quantitatively measure a worm (forced) velocity within a few minutes and *a priori* in a much more reproducible way than what can be achieved by observation of freely moving worms. Performing electrotactic runs with 2 or more worms should allow discriminating between slow and fast worms. Therefore, using such a simple electrotaxis apparatus gives an efficient way to serial sort worms based on their locomotor fitness.

**Figure 3:**
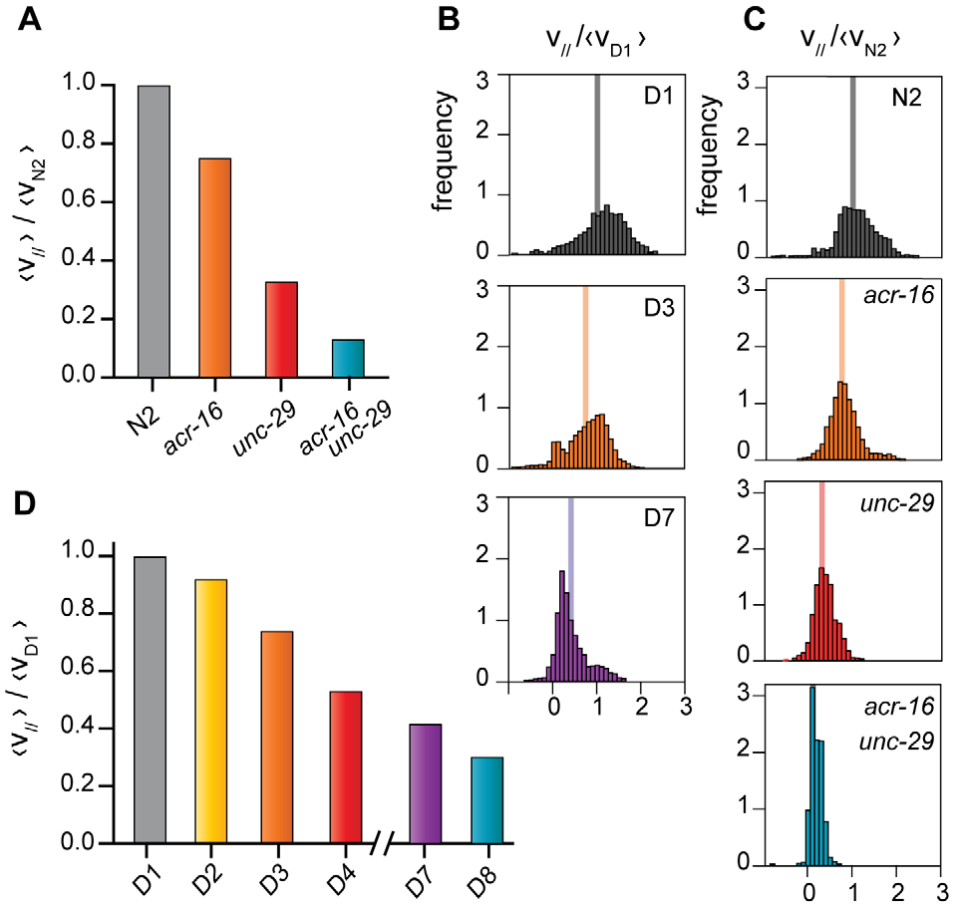
Comparative analysis of mutant worms and chronological aging effects on forced locomotory abilities. (A) *acr-16* (v_//_ = 80 µm/s±40 µm/s, N = 28), *unc-29* (v_//_ = 35 µm/s±34 µm/s, N = 26) and *unc-29*;*acr-16* mutants (v_//_ = 15 µm/s±19 µm/s, N = 22) exhibit reduced velocity when compared to the control population (N2, v_//_ = 110 µm/s±50 µm/s, N = 28) in successive electrotactic runs. Errors are computed as standard deviations. (B) Velocity histograms for aging populations (cf. D). (C) The histograms of v_//_for mutant worms are significantly different (p<0.05, Fisher test). (D) Populations of worms show a decrease of the average velocity as they get older, from the 1^st^ day (D1) to the 8^th^ day (D8). Here, the average parallel velocity at Day 1, <v_D1_>  = 120 µm/s is taken as a reference. Number of worms: D1/N = 17; D3/N = 15; D7/N = 6. The normalized average velocity is indicated by a vertical line on the histograms (B, C).

**Figure 4:**
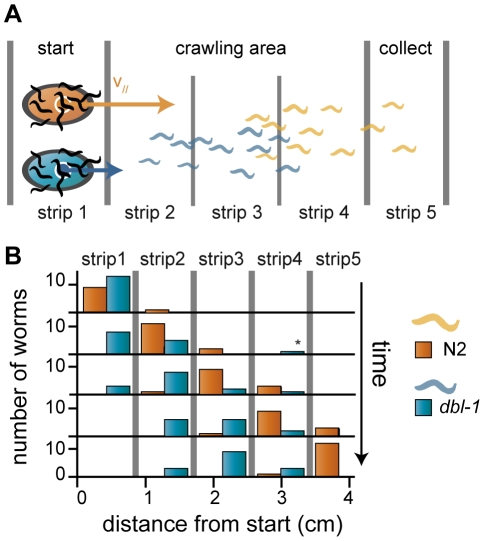
Population sorting I. (A) Principle of population sorting. (B) Sorting in action. We conducted a sorting experiment with a mix of 15 wild-type and 15 *dbl-1* worms. The number of wild-type worms (orange) and *dbl-1* mutant worms (blue) are shown as a function of time and space. We divided the observed area into 5 slices of equal size and computed the number of worms of each strain at different time points (every 2 minutes). Progressively, the wild-type worms separate from the initial mix. The final strip contains only wild-type worms, while, the 2^nd^ and 3^rd^ stripes contain only *dbl-1* mutant. The experiment was repeated three times. See also Movie S4.

**Figure 5:**
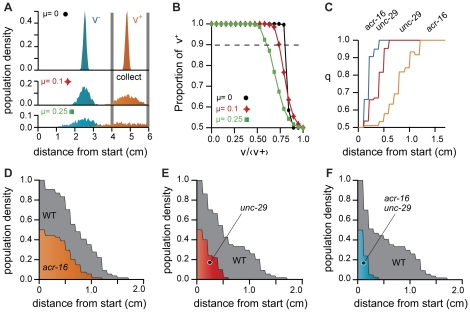
Population sorting II. (A) We numerically computed the histograms of the distance traveled by a fictitious population of 1000 worms assuming a Gaussian velocity distribution for each worm with an average parallel velocity given by v_0_(1+gnoise(µ)) and a standard deviation of 50 µm/s. The resulting population has 1000 worms with normally distributed averaged velocity and displays larger intra-population variability for larger µ. We then compared two populations with different v_0._ Wild-type worms (orange) are moving at <v^+^>  = 200 µm/s and slow worms (blue) are moving at <v^−^>  = 100 µm/s. (B) The same principle allows to compute the proportion of wild-type worms (v = 200 µm/s) in the collect area (>4 cm from start) as a function of the ratio of the average velocity of the two populations. As expected, population with similar dynamics and intra population variability of velocities (larger µ) decrease the sorting efficiency. (C) Using the experimental data displayed on Fig. 3 we computed the distribution of worms position at time τ = 100s. Calling f_1_(x) and f_2_(x) the two position distributions, the fraction of population 1 over population 2 as a function of the distance traveled is given by 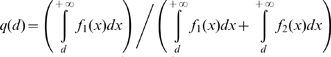.When starting from a fictitious 50% mix of wild-type and any of the mutant strains *acr-16*, *unc-29* or *unc-29;acr-16* (see text), the sub-population is quickly enriched in wild-type worms (faster worms) as the distance of capture increases. Ideally, capturing worms as far as possible from the starting point ensure a perfect sorting. (D,E,F) However, since not all worms move at their maximum velocity during electrotaxis, there is a tradeoff between the degree of separation and the total number of worms that can be captured. Population densities decrease with the distance to start.

In the following, we explore and validate this approach on three biologically relevant examples: (1) the quantitative comparison between wild-type and mutants displaying altered locomotion (Fig. 3, Fig. 4), (2) the effects of aging on the locomotory rate of worms (Fig. 3) and (3) the actual separation of a mix of two worm strains (Fig. 4, Fig. 5). We then discuss the potential of this method.

### Wild-type vs slow worms

*C. elegans* body wall muscles have two functional acetylcholine receptors activated by levamisole and nicotine respectively. UNC-29 is a subunit of the levamisole sensitive receptor [17], [18] and ACR-16 is a subunit of the nicotine sensitive receptor [19]. Both *unc-29* and *unc-29;acr-16* mutants have been shown to move at a slower rate with uncoordinated phenotype, the double mutant being less active [19]. However, it is difficult to quantitatively measure the velocity of such phenotypes because they only move occasionally. We conducted several electrotaxis runs on wild-type *C. elegans*, the single mutants *unc-29* and *acr-16*, and the double mutant *unc-29*;*acr-16*. All mutant strains were reactive to the presence of an electric field. They showed a directed locomotion allowing us to measure their velocity precisely. Wild-type worms were the fastest worms (v_//_ = 110 µm/s), followed by *acr-16* (v_//_ = 80 µm/s), *unc-29* (v_//_ = 35 µm/s) and finally *unc-29*;*acr-16* mutants (v_//_ = 15 µm/s). This simple experiment confirms that the double mutant has a much more pronounced phenotype, in good agreement with the fact that *unc-29* and *acr-16* mutations impair both acetylcholine receptors. Moreover, we were able to discriminate between *acr-16*, *unc-29* and wild-type worms on the sole base of their velocities difference.

### Quantitative influence of aging on locomotion

Locomotion has been proposed as a qualitative way to score for aging [20], [21]. The worm electro-tactic abilities relationship with age has not been studied in details yet, but a recent experiment suggested that all larvae stages and young adult worms were responsive to an electric field [22]. Here, we subjected worms of increasing ages, from young adult at day 1 (*D1*) to older worms at day 8 (*D8*), to electrotaxis trials. Worms of all ages were responsive and run directionally, although older worms tend to follow less straight trajectories. The average worm velocity decreased with age by 70% between day 1 and day 7 (Fig. 3). Hence, directed locomotion by electrotaxis gives a quantitative, user-independent indicator of physiological aging. An interesting follow-up, would be to test whether this physiological aging is related to lifespan and how it correlates with aging-related muscle degeneration.

The two previous examples evidence how electrotaxis can be used to perform quantitative measurements of single worm velocity. Such measurements can then be used to identify a given phenotype or to get a quantitative estimate of a worm physiological state. Although it is a quantitative method, it remains time consuming to perform such experiments using one worm at a time. An alternative approach is to force many worms to race against each other.

### Worms self-sorting

If all worms do not move at the exact same speed, the population will spread on the gel, creating a phenotypical gradient from slow to fast worms (Fig. 4a). In other words, electrotaxis could be used to spatially sort worms according to their velocity, very much like DNA is sorted by molecular weight during electrophoresis. Sorted population of worms can be recovered after the trial by selecting worms at a given distance from the original starting point. As a proof of concept, we tested this method on a population mix of wild-type worms and *dbl-1* mutants (Movie S4). DBL-1 is the TGF-β-related ligand for the Sma/Mab pathway [22]. Loss of DBL-1 activity results in smaller animals which makes them easy to distinguish from wild-type animals. Interestingly those worms turned out to be slower than wild-type worms. Figure 4b shows the number of wild-type and *dbl-1* worms at different time and position along the gel. All worms that have traveled at least 4 cm after 8 min were wild-type worms, thus demonstrating in practice the efficiency of self sorting by electrotaxis. We therefore achieve to sort the initially mixed population. Since population separation is the result of differential locomotory rate between the worms, the variability of velocities within a population can affect the sorting process. We checked numerically that indeed the sorting efficiency is decreased by increasing the velocity variability between worms of the same population as shown in figures 5a and 5b. Finally, to try realistic velocities distribution, we used our experimental data on mutants (reported on Fig. 3) and analyzed how a 50/50 mix of two populations of such worms would self-sort. We computed the relative enrichment in fast worms (wild-type) as a function of the distance at which worms would be captured after a given fixed time (Fig. 5c–f). In every case, populations were quickly enriched into the fastest worm (wild-type, WT).

## Discussion

Taken together, our experiments show that electrotaxis can be used to quantitatively measure the speed of single worms and to sort a population based on its worms' velocities within only a few minutes. Physical sorting only depends on the distance worms are able to crawl in a given amount of time. Whereas, it is possible to design complex electrotaxis setup [16], [23], we shall insist that a simple, commercial electrophoresis system is sufficient to physically sort the faster worms from a population. A vision system placed above the electrophoresis box is only needed to get quantitative measurements.

A recent study proposed a micro-fluidic approach to sort worms by electrotaxis according to their swimming speed differences [23]. Although this approach was interesting it suffered from two intrinsic limitations: (i) the difficulty to use it with a very large number of worms – high throughput micro-fluidics are highly demanding – and (ii) its small dimensions, since it is always more efficient – and easier – to use a large device for sorting. Indeed, the spatial resolution increases with the length on which worms are allowed to run. Finally, it is important to note that catching the worms back from the micro-fluidic channel was an apparently unsolved challenge. Our elementary method overpasses those limitations. In particular, using a macroscopic electrophoresis setup increases the resolution of the sorting process. The sorting resolution is limited by the size of the electrotaxis gel and by the number of worms. As a matter of fact, two (populations of) worms with well defined velocity (Fig. 5a) are separable if the length of the gel is longer than δ v_max_/Δv, where Δv is the relative difference of their average speed, v_max_ the velocity of the fastest worm and δ the typical size on which worms are captured afterwards. With δ  = 1 cm, v_max_  = 150 µm/s and a gel of 10 cm, the speed resolution is Δv  = 15 µm/s, which is smaller than the velocity standard deviation within a population. However, the effective resolution is lowered by the variability of velocities within a population (Fig. 5a,b). If the two populations are not well separated after one run, isolating a sub-population and performing another electrotaxis race will allow further separation of this sub-population. Iterating this process will increase the degree of sorting of the sub-population and remove almost all the slow worms (mutants) after a few trials. This method allows increasing the resolution but at the expense of decreasing the number of worms that can be sorted and collected. Since even isogenic population exhibits a rather large variability of their navigation velocity, this method could be used to prepare population samples with well defined locomotion abilities and presumably similar physiological state. Uncoordinated worms or worms defective in sensing the field should also be separable from wild-type worms since they will remain near the starting area.

We demonstrated here the practical potential of our method in separating a large number of worms to select for the desired phenotype as long as it is related to locomotion, which is very quite often the case for nematodes [1]. It may be efficiently combined with a worm sorter, transcriptomics, RNAi, or biochemical analysis, to correlate the physiological state of the worms with the expression level of specific genes. We also showed how gel-electrotaxis assay can be used to get quantitative data of the dynamical behavior of worms within a few minutes only. *C. elegans* is a growing model for neurodegenerative diseases and diseases linked with muscle degeneration. These dysfunctions affect locomotory behavior. We anticipate that gel-electrotaxis serial sorting combined with high-throughput screening of bioactive molecules could help to find innovative therapeutic strategies to these diseases.

## Methods

### Strains

We used wild-type strain (N2), *dbl-1* mutants and the mutants *acr-16*(*ok789*), *unc-29*(*x29*), *unc-29*(*x29*); *acr-16*(*ok789*) obtained from the laboratory of J.-L. Bessereau (ENS, INSERM U 789). *C. elegans* worms were developed at 15°C and then at 25°C during adulthood. They grew on NGM plates seeded with *E. coli* OP50.

### Electrotaxis assay

In each experiment approximately 10–15 worms were selected from a cultivation plate of a synchronized population of adults and rinsed with M9 buffer solution. They were then transferred on an agar gel in a drop of M9. The agar gel was composed of: de-ionized water, 2% of Bacto-Agar, glycerol (3.7 mL of glycerol 60% for 1 L), NaCl (0.250 mmol/L) as previously described in [16]. The gel was cast by pouring a first layer of agar and adding a PDMS (polydimethylsiloxane) block onto it so that it will shape the future cavity where nematodes will crawl (6×8 cm). A second layer of gel was then poured around the PDMS block. Once solidified the PDMS block was removed. The resulting agar pad was then placed in an electrophoresis box filled with a buffer. It was composed of de-ionized water, glycerol (3.7 mL of glycerol 60% for 1 L) and NaCl (0.250 mmol/L) as previously described in [16]. We used a PS305 electrophoresis power supply (APELEX, France) and the Wide Mini-Subtm Cell electrophoresis box (Biorad, USA).

### Image analysis

Experiments were imaged with a 6.6 Mpixels CMOS monochrome camera (Pixelink) with a close focus zoom lens 10X (13×130 mm FL, Edmund Optics Ltd, UK). We used a white, bright field/dark field ring light (Edmund Optics Ltd), to enhance the contrast. Since the worm trajectories are ideally straight, image analysis was straightforward. Trajectories of worms were computed from images by using successively ImageJ http://rsbweb.nih.gov/ij/ [24] and its *analyze particles* plug-in to detect worms position at every time step (1 frame per second), the GNU Octave software (http://www.gnu.org/software/octave/) and finally Igor Pro (Wavemetrics, USA) to construct trajectories, perform data manipulation and compute statistical tests. Velocities were computed by averaging the displacement of the center of mass of nematodes over 10 frames (10 s).

### Numerical analysis

The histograms and enrichment proportion displayed in Figure 5a and 5b were numerically computed. We computed the evolution with time of the position of 1000, worms assuming that every worm has a speed set by a Gaussian distribution with a standard deviation of 50 µm/s (Fig. 4c and 4d). Positions were updated every second during 240 s. To increase the variability of the total population, we allowed the average velocity of single worms, v_s_, to vary from one worm to another by adding a Gaussian noise: v_s_ = v_0_(1+g(µ)), where g is a function that returns a random value from a Gaussian distribution of standard deviation µ. The resulting population has1000 worms, with normally distributed averaged velocity and display larger variability for larger µ.

## Supporting Information

Movie S1 Worms performing electrotaxis. Worms randomly placed at the surface of an agar gel are able to sense the presence of an electric field. They crawl with straight trajectories, punctuated by collisions, reversals and other rare navigation behaviors. This figure displays the trajectories of 14 worms during a short electrotactic run. Red circles indicate the worms' starting positions.
(WMV)

Movie S2 Reorientation during electrotaxis. Worms sometimes display a reorientation behavior, with omega-like reversal before resuming their trajectory. On this specific example, it takes a few seconds for the worm to reorient itself. Such behavior occurs rarely in the presence of an electric field.
(WMV)

Movie S3 Sudden reversal of the electric field. Just after a sudden decrease of the electric field, worm abruptly stop its motion, and makes a few omegas reversals. When the electric field is reversed, worms move also in a reversed direction. The worm has been colored in red, green or blue to illustrate the different phase of its adaptation to an electric field reversal. The scale is given by the size of the worm (typically 1 mm) and there are 10 seconds between each picture. This suggests that variable electric field (in time and direction) could also be used to serial sort population of worms based on their electrotactic dynamics.
(WMV)

Movie S4 Worms sorting: proof of concept. This movie illustrates the separation of a population composed of a mix of 15 wild-type worms and 15 *dbl-1* mutants which are smaller and slower (see also Fig. 4b ) than wild-type. Wild-type worms (larger ones) are faster and progressively separate away from the *dbl-1* mutants. The field of view is typically 5 cm long and the movie lasts for ∼4 minutes.
(WMV)
